# Anti-proliferative and pro-apoptotic effects of curcumin on skin cutaneous melanoma: Bioinformatics analysis and *in vitro* experimental studies

**DOI:** 10.3389/fgene.2022.983943

**Published:** 2022-09-12

**Authors:** Long Li, Shuwen Lu, Chao Ma

**Affiliations:** ^1^ Department of Ophthalmology, The First Affiliated Hospital of Zhengzhou University, Zhengzhou, China; ^2^ Department of Ophthalmology, the First Affiliated Hospital of Henan University of Chinese Medicine, Zhengzhou, China

**Keywords:** curcumin, skin cutaneous melanoma, biomarker, proliferation, apoptosis, migration

## Abstract

**Objective:** To reveal the potential mechanisms of curcumin for the treatment of skin cutaneous melanoma (SKCM) and its identify novel prognostic biomarkers.

**Methods:** We searched the Cancer Genome Atlas and Traditional Chinese Medicine Systems Pharmacology database for the data on SKCM and curcumin. We conducted data analysis using R and online tools. The propagation and migration of SKCM cells were assessed with CCK-8 and scratch wound assays, respectively. We assessed apoptosis by TUNEL assay and western blot.

**Results:** The survival analysis revealed that the mRNA expressions of *DPYD*, *DPYS*, *LYN*, *PRKCQ,* and *TLR1* were significantly related to a favorable overall survival in SKCM patients. Additionally, the mRNA expression level of *DPYD* was associated with *GPI*, *LYN*, *PCSK9*, *PRKCQ,* and *TLR1* mRNAs. GSEA results showed that the prognostic hub genes were augmented with ultraviolet, apoptosis, and metastasis. Curcumin expressed proliferation and migration of SK-MEL-1 cells (*p* < 0.05), and induced apoptosis (*p* < 0.05) significantly.

**Conclusion:** Curcumin may have potential therapeutic effects in SKCM by inhibiting cell proliferation and migration and inducing apoptosis by regulating oxygen-related signaling pathways. The hub genes might be identified as novel biomarkers for SKCM.

## 1 Introduction

Skin cutaneous melanoma (SKCM) is one of the most vigorous and fatal skin cancer types. The worldwide incidence of SKCM increases faster annually than any other cancer (Ali et al., 2013). The latest research shows that about 95,830 new cases of SKCM *in situ* have been reported in the United States in 2019 (Siegel et al., 2019). Ultraviolet (UV) radiation may be the main environmental risk factor (Gilchrest et al., 1999). Although the pathogenesis and diagnostic methods of diseases have shown great progress, the morbidity and mortality of SKCM have increased over the past 50 years in developed countries (Lu et al., 2018). Moreover, the treatment strategies that are currently available for metastatic melanoma have shown a relatively poor rate of success, and most newly developed anti-melanoma treatments are associated with severe adverse reactions (Kalal et al., 2017; Kozar et al., 2019; Luther et al., 2019). For these reasons, people have started to gain interest in natural compounds. It has been found that phytochemicals have demonstrated anti-proliferation, apoptosis promoting, anti-invasion, and anti-angiogenesis properties in mouse models and melanoma cell lines, without obvious toxicity (Fontana et al., 2019).

Curcumin is a polyphenolic compound, and curcumin is derived from turmeric (Curcuma longa), as its primary bioactive component (Pisano et al., 2010). It is a dietary spice made from the rhizome of Curcuma longa and is commonly used in curry powder as well as for centuries in traditional Chinese medicine (Maheshwari et al., 2006; Yu et al., 2010). Research has identified that curcumin has various therapeutic properties via different biological functions and pharmacological effects. These therapeutic properties include anti-inflammatory, antioxidant, immunomodulatory, antimicrobial, anti-ischemic, anti-cancer, and antirheumatic activities (Sahebkar, 2010, 2013; Mirzaei et al., 2016; Momtazi et al., 2016; Pulido-Moran et al., 2016; Kunnumakkara et al., 2017). Curcumin can induce endoplasmic reticulum stress in SKCM by inhibiting classic signaling pathways, which included nuclear factor kappa B (NF-κB), signal transducer and activator of transcription 3 (STAT3), Akt/Mtor, and Wnt/β-catenin, the expression of reactive oxygen species (ROS)thus the enhancement of oxidative stress injury has been increased (Bakhshi et al., 2008; Liao et al., 2017; Siwak et al., 2005; Zhang et al., 2015; Zheng et al., 2004). However, no systematic study on the mechanism, target, and effect of curcumin in the course of SKCM has been published thus far.

Therefore, we used the resources in traditional Chinese medicine and tumor databases to analyze what role curcumin plays in the treatment of SKCM. Specifically, we aimed to identify the target genes of curcumin acting on SKCM and analyze whether these genes are related to pathogenesis, staging, and prognosis. Furthermore, the effects of varying concentrations of curcumin on proliferation, migration, and apoptosis of SKCM cells were assessed *in vitro*. The results of this study were the basis for future studies on the effects of curcumin on SKCM.

## 2 Materials and methods

### 2.1 Detection of potential target genes

We obtained the molecular formula of curcumin from the Traditional Chinese Medicine Systems Pharmacology Database and Analysis Platform (TCMSP, https://old.tcmsp-e.com/tcmsp.php, accessed on 25 June 2021) (Ru et al., 2014). The effectiveness of target genes was identified from the PharmMapper Server (http://www.lilab-ecust.cn/pharmmapper/, accessed on 25 June 2021) (Liu et al., 2010; Wang et al., 2016; Wang et al., 2017) through Druggable Pharmacophore Models. Besides, we obtained the SKCM-related target genes and all of the protein-coding genes from the GeneCards Human gene database (https://www.genecards.org/, accessed on 25 June 2021) (Harel et al., 2009; Stelzer et al., 2016). Thus, the common target genes of curcumin and SKCM were identified.

### 2.2 Gene ontology and kyoto encyclopedia of genes and genomes analyses

We conducted the GO and KEGG analyses based on these common target genes. Providing data about the gene expression of common target genes. The GO and KEGG analyses were conducted to elucidate which mechanisms were applied by curcumin in the treatment of SKCM using the KOBAS 3.0 web server (http://kobas.cbi.pku.edu.cn/index.php, accessed on 30 June 2021) (Wu et al., 2006; Xie et al., 2011) and STRING v11.0 (https://string-db.org/, accessed on 30 June 2021) (Szklarczyk et al., 2019). Additionally, the Biological Networks Gene Ontology (BiNGO) (Maere et al., 2005), a GO function analysis tool, was applied to predict the functionality of common target genes.

### 2.3 Establishing the Protein-Protein Interaction network

A PPI network provides systematic visual data on the relationships between drugs, target genes, and proteins. We obtained the data from the STRING protein query and utilized data to construct the PPI network. Medium confidence of 0.400 was selected as the threshold in the analysis. Some nodes that were disconnected from each other were not displayed. Cytoscape software 3.6.1 was used to visualize the PPI network.

### 2.4 The construction of Genetic Interaction network

A GI network shows the complex interaction between genes of interest, and it was generated in GeneMANIA (https://genemania.org/, accessed on 30 June 2021) (Warde-Farley et al., 2010). The common target genes were used as query terms and then the predicted ones were shown simultaneously.

### 2.5 Survival data preparation

We obtained the Cancer Genome Atlas (TCGA) data and survival rate/time data, including clinical information (ID, age at index, gender, race, vital status, tumor stage, treatment, and the mRNA expression of common target genes of SKCM patients) from OncoLnc (http://www.oncolnc.org/, accessed on 25 June 2021) (Anaya, 2016) and TCGA (https://cancergenome.nih.gov/, accessed on 1 July 2021). Briefly, all common target genes were registered in the database, then the patients with SKCM were categorized half based on the expression of every gene, and as a result, the survival data of these SKCM patients was obtained. Ultimately, the common target genes related to overall survival (OS) were identified as hub genes.

### 2.6 Survival analysis

We used the 50% limitation as standard for each hub gene’s mRNA to divide the patients into groups with high- or low expression. Based on OS, the log-rank test and Kaplan-Meier estimator were applied in the survival analysis to calculate the log-rank *p*-value and identify the OS of hub genes. Subsequently, a Cox regression analysis was conducted to identify any associations between clinical information and the risk score, for which a nomogram was produced. R v3.6 was used to create the survival curves and nomogram.

### 2.7 mRNA expression levels and correlation analyses

The Gene Expression Profiling Interactive Analysis (GEPIA: http://gepia.cancer-pku.cn/, accessed on 1 July 2021) dataset (Tang et al., 2017) was applied to create a boxplot in which the hub gene mRNA’s expression levels were demonstrated. We calculated the mRNA expression levels through the retrieved TCGA data. Furthermore, we defined the expression of hub genes as high and low based on the median value. The high expression group referred to patients with expression values that were higher than the median values of the specific hub genes. Besides, the low expression group referred to patients with expression values that were lower than the median values of the specific hub genes. R v3.6 was used to perform Pearson correlation coefficient analysis by which the co-expression relationship among hub genes was assessed.

### 2.8 Gene Set Enrichment Analysis

GSEA (http://software.broadinstitute.org/gsea/index.jsp; accessed on 1 July 2021) (Subramanian et al., 2005) was conducted to identify which potential mechanisms were responsible for the effect that the risk score has on SKCM prognosis. The Molecular Signatures Databases (MSigDB) c2 (c2. cp.kegg.v6.1. symbols.GMT) and c5 (c5. all.v6.1. symbols.GMT) were used to investigate the crucial functions and pathways that could affect SKCM on the basis of prognosis-related hub gene mRNAs. We defined the significance as a nominal *p*-value < 0.01 and false discovery rate (FDR) < 0.25 for the sets of enrichment genes in the GSEA. The nine most significant gene sets were selected for this study and six of the eight prognosis-related genes (*DPYD*, *GPI*, *LYN*, *MMP2*, *PRKCQ,* and *TLR1*) were included in the GSEA due to the limitation of the dataset.

### 2.9 Cell line and drugs

The human SKCM cell line (SK-MEL-1) was retrieved from Procell Life Science & Technology (Wuhan, China). The curcumin was obtained from Solarbio Life Sciences (Beijing, China). The cells were cultured in Dulbecco’s Modified Eagle Medium (DMEM) added with 20% FBS and then incubated at 37°C.

### 2.10 Cell proliferation assay

We used a Cell Counting Kit-8 (CCK-8) assay (Beyotime Biotechnology, Shanghai, China) to measure the spectrophotometric absorbance, with which the proliferation of cells at 24 h was estimated. The SK-MEL-1 cells were cultured on plastic 96-well culture plates at a concentration of 5 × 10^3^ cells/well. All experiments were performed as instructed by the manufacturer.

### 2.11 Cell migration assay

Cell migration was detected through a scratch wound assay. The SK-MEL-1 cells confluence in culture plates was scratched with a sterile pipette tip to produce a space free of cells. Serum-free DMEM was used to first rinse all the cells and then they were photographed to document the width of the wound at 0 h. Then, we used the serum-free medium to culture the negative control group of cells for up to 24 h, and the other two groups were treated with 20 and 30 μM curcumin, respectively. Photographs of the marked wound location were taken again at 6, 16, and 24 h to measure the migration of cells.

### 2.12 TUNEL assay

The rate of apoptosis in cultured SK-MEL-1 cells was measured with the One Step TUNEL Apoptosis Assay Kit (Dalian Meilun Biotechnology, Dalian, China) as described by the manufacturer’s instructions. SK-MEL-1 cells cultured in plastic 6-well culture plates were treated with proteinase K (20 μg/ml) and stained as recommended. The apoptotic index of cells was calculated by observing the TUNEL-positive cells in six fields that did not overlap under ×200 magnification.

### 2.13 Western blot

Cell apoptosis was also evaluated on protein level by western blot. The primary antibodies (GAPDH, Bcl-2, Cleaved Caspase 3, and Bax) were obtained from Cell Signaling Technology Inc. (MA, United States). The extracts of protein were stored at −80°C until use. Identical quantities of denatured protein were exposed to 10% SDS-PAGE and the separated proteins were placed on polyvinylidene difluoride (PVDF) membranes (Solarbio Life Sciences, Beijing, China). An LI-COR automatic chemiluminescence image analysis system was used to visualize the protein bands. The Odyssey Fc Imaging System was used to quantify the Western blot signals.

### 2.14 Statistical analyses

**FIGURE 1:**
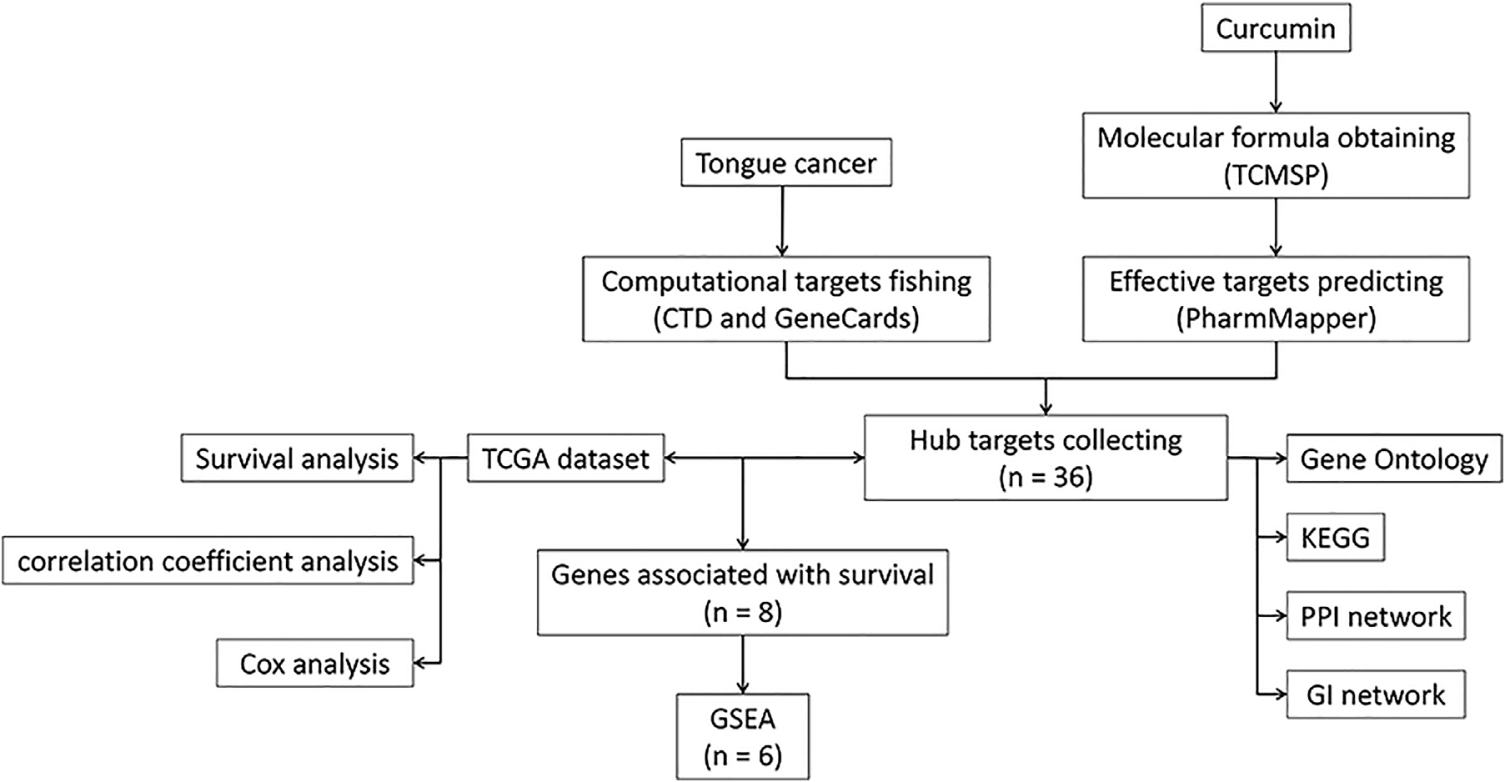
A workflow diagram. TCMSP, Traditional Chinese Medicine Systems Pharmacology Database, and Analysis Platform; KEGG, Kyoto encyclopedia of genes and genomes; PPI, Protein-Protein Interaction; GI, Genetic Interaction; TCGA, The Cancer Genome Atlas; GSEA, Gene Set Enrichment Analysis.

We used R v3.6 to obtain the correlation plot, survival curves, nomogram, and visualization of data. A *p*-value < 0.05 was deemed statistically significant. A workflow diagram is shown in Figure 1. GraphPad Prism (GraphPad Software, San Diego, United States) was used to perform one-way and two-way ANOVA.

## 3 Results

### 3.1 The identification and functional analyses of target genes

**FIGURE 2:**
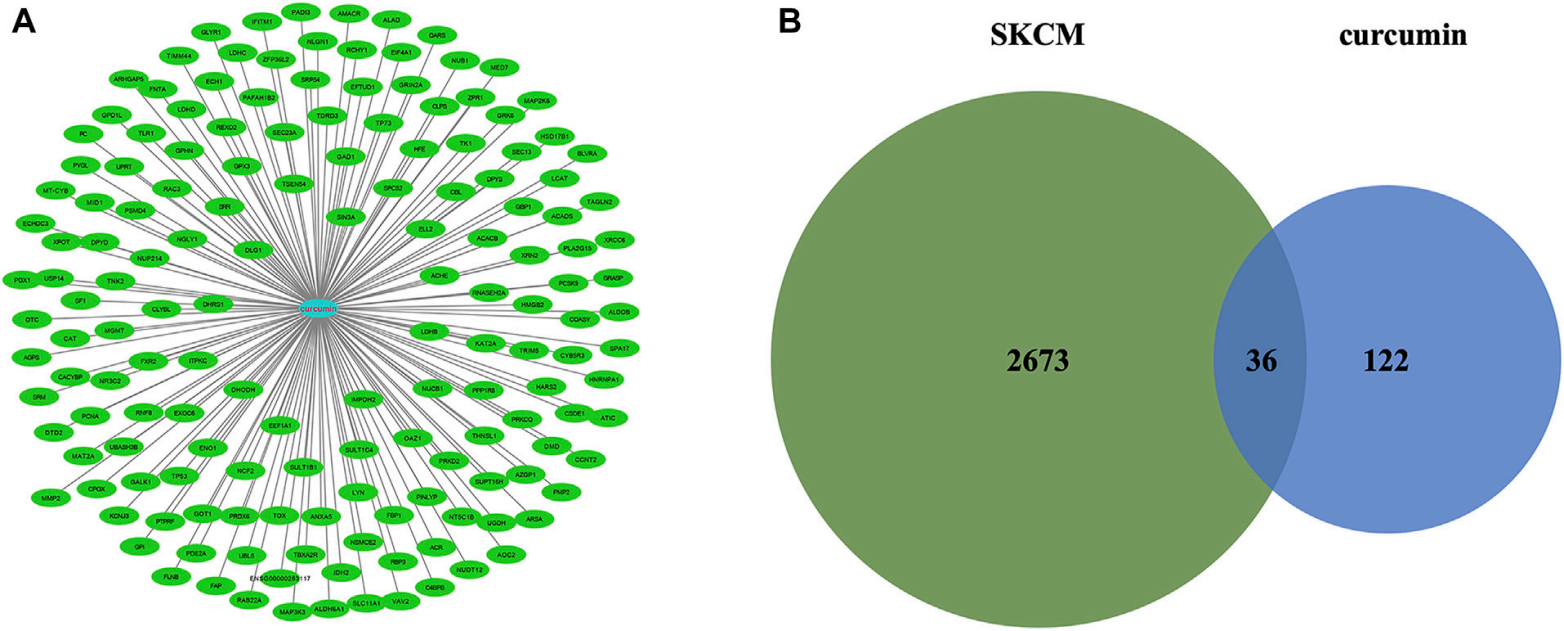
**(A)** Targets for curcumin. **(B)** Venn diagram summarizing differentially expressed targets for SKCM and curcumin. SKCM, skin cutaneous melanoma. The data are derived from PharmMapper.

The molecular formula of curcumin was obtained from TCMSP and 158 human target genes were collected (Figure 2A), of which 36 were identified as common targets (Figure 2B). The significant (the cut-off criterion for statistic difference was corrected to *p* < 0.0001) GO categories are shown in Supplementary Table S1. The common target genes were mainly enriched in the cytoplasm (GO:0005737), catabolic process (GO:0009056), response to endogenous stimulus (GO:0009719), and other terms. The KEGG analysis showed that the hub genes were mainly enriched in carbon metabolism (hsa01200), metabolic pathways (hsa01100), and NF-κB signaling pathway (hsa04064), and other associated pathways (Supplementary Figure S1). These results were consistent with the BiNGO outcomes (Supplementary Figure S2).

### 3.2 The construction of PPI and GI network

The PPI network was constructed using the STRING online tool, and the Cytoscape 3.6.1 software was used for visualization. Identification of the most significant genes using a network constructed from common target genes (Supplementary Figure S3A). As shown in Supplementary Figure S3A, the tumor protein 53 (*TP53*), catalase (*CAT*), and enolase 1 (*ENO1*) were evidently at the PPI network’s center. The GeneMANIA online tool was used to construct the GI network, which shows the interaction among the 36 common target genes and predicted genes (Supplementary Figure S3B).

### 3.3 Survival analysis

**FIGURE 3:**
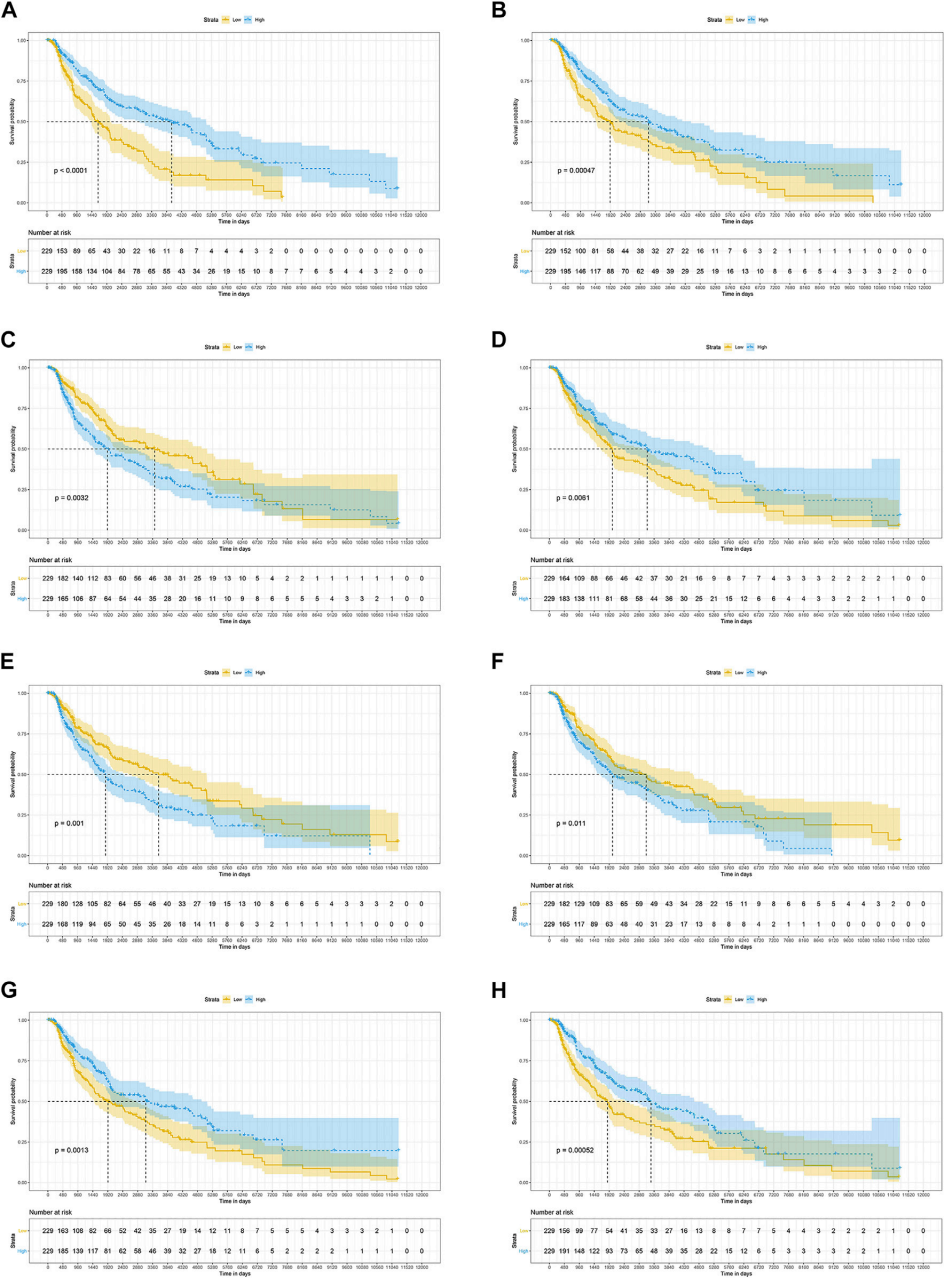
The prognostic significance of common targets for the OS of SKCM patients **(A–H)** Kaplan-Meier survival curves for all SKCM patients based on DPYD **(A)**, DPYS **(B)**, GPI **(C)**, LYN **(D)**, MMP2 **(E)**, PCSK9 **(F)** PRKCQ **(G)**, and TLR1 **(H)** expression (*n* = 458). OS, overall survival; SKCM, skin cutaneous melanoma.

**FIGURE 4:**
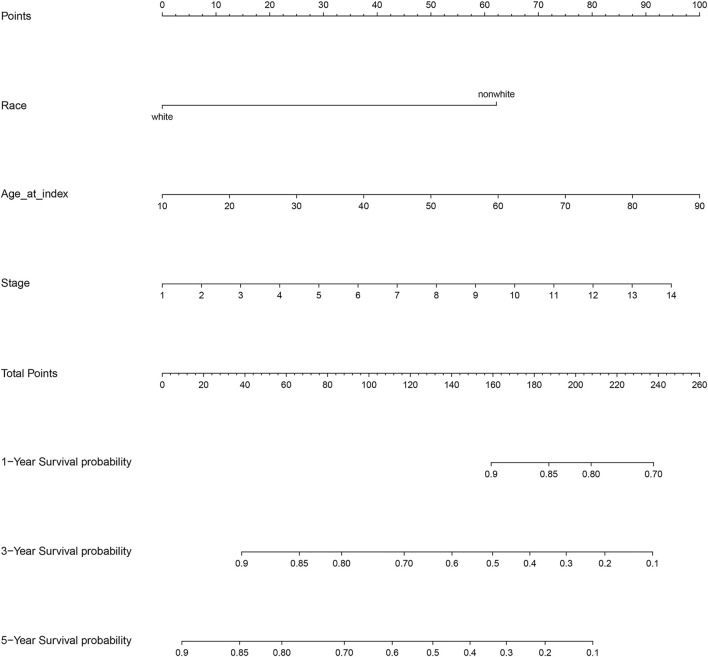
Nomogram for the relationship between clinical data and risk score. Stage 1 for I/II nos, 2 for 0, 3 for I, 4 for I a, 5 for I b, 6 for II, 7 for II a, 8 for II b, 9 for II c, 10 for III, 11 for III a, 12 for III b, 13 for III c, and 14 for IV.

The results of the log-rank test and Kaplan-Meier estimator indicated that the following eight out of 36 common target genes were significantly associated to the OS of SKCM patients: matrix metallopeptidase 2 (*MMP2*, *p* = 0.001), toll like receptor 1 (*TLR1*, *p* = 0.00052), dihydropyrimidine dehydrogenase (*DPYD*, *p* < 0.0001), proprotein convertase subtilisin/kexin type 9 (*PCSK9*, *p* = 0.011), protein kinase C theta (*PRKCQ*, *p* = 0.0013), glucose-6-phosphate isomerase (*GPI*, *p* = 0.0032), dihydropyrimidinase (*DPYS*, *p* = 0.00047), and the LYN proto-oncogene, Src family tyrosine kinase (*LYN*, *p* = 0.0061) (Figure 3). Based on the clinical information of SKCM patients, race (*p* = 0.0072), age at index (*p* < 0.0001), and tumor stage (*p* < 0.0001) were all correlated to OS. Figure 4 shows the generated nomogram and the c-index of this model was 0.669.

### 3.4 mRNA expression levels and correlation analyses

As shown in boxplots, significant differences were discovered between the mRNA expression levels of hub genes found in normal tissues and SKCM tissues. Furthermore, the mRNA expression of *DPYS*, *GPI*, *TLR1*, *PRKCQ,* and *LYN* (Supplementary Figures S4B–D,G,H) in SKCM tissues were greater compared to those of normal tissues, of which the difference was statistically significant in *GPI*, *PRKCQ,* and *LYN* (all *p*-value < 0.01, Supplementary Figures S4C,G,H). In contrast, the mRNA expression of *DPYD*, *MMP2,* and *PCSK9* in SKCM tissues was lower in comparison to that of normal tissues (Supplementary Figures S4A,E,F), of which the difference was only statistically significant in *MMP2* (*p*-value < 0.01, Supplementary Figure S4F).

The correlation between the mRNA expression levels of hub genes was determined by Pearson correlation coefficient analysis. The results have shown that the mRNA expression level of *DPYD* was correlated with most of the hub gene mRNAs (*GPI*, *LYN*, *PCSK9*, *PRKCQ,* and *TLR1*) (all *p*-value < 0.01, Supplementary Figure S5 ; Supplementary Table S2).

### 3.5 GSEA analysis

GSEA was performed to calculate an enrichment score (ES) by going through the list of genes. The green line, representing a running-sum statistic, was enhanced when a gene was part of the gene set and decreased when it was not. A positive ES indicated enrichment of the gene set at the top of the ranking list and a negative ES indicated enrichment at the bottom. The horizontal bar’s red part and blue part represented positive and negative ES, respectively.

The results of the GSEA indicated that *DPYD* was primarily enriched in apoptosis, the JAK/STAT and MAPK signaling pathways, and the response to UV and cell adhesion functions (Supplementary Figure S6). For *GPI*, the AKT1 signaling pathway and the response to ultraviolet, glucose metabolism, oxidative phosphorylation, and electron transport chain were mainly enriched (Supplementary Figure S7). *LYN* was enriched in MAPK, JAK/STAT, and apoptotic-related signaling pathways. Additionally, *LYN* was also associated with DNA damage by UV and cell adhesion (Supplementary Figure S8). According to the results, *MMP2* was highly related to cell migration and adhesion, apoptosis, and skin cancer progression (Supplementary Figure S9). As demonstrated, *PRKCQ* was mainly enriched in the JAK/STAT and MAPK signaling pathways, as well as apoptosis, DNA damage by UV, and cell adhesion (Supplementary Figure S10). *TLR1* was mainly associated with the Toll-like receptor, JAK/STAT and AKT1 signaling pathways, and UV-induced apoptosis and DNA damage (Supplementary Figure S11).

Overall, the hub prognosis-related genes were mainly associated with cell adhesion and UV-related functions and participated in JAK/STAT and MAPK signaling pathways. Notably, *MMP2* was associated with skin cancer progression.

### 3.6Curcumin inhibits SK-MEL-1 proliferation

**FIGURE 5:**
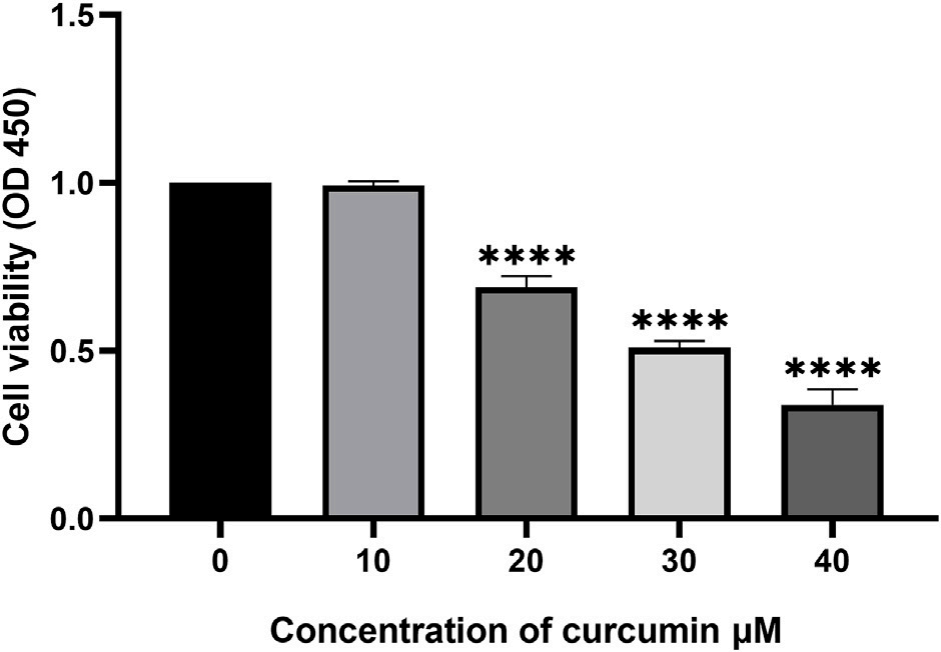
Study of cytotoxicity by using CCK-8 assay. The SK-MEL-1 cells were treated with curcumin in different concentrations. The cell proliferation was significantly decreased under the stimulation of 20 and 30 μM curcumin compared to that of 10 μM ***p < 0.001 and ****p < 0.0001, one-way ANOVA.

The anti-proliferative effect of curcumin on SK-MEL-1 cells was evaluated *in vitro* by a CCK-8 assay. SK-MEL-1 cells were treated with different concentrations of curcumin, and the results showed that the inhibition of cell proliferation was more pronounced at 30 than at 20 μM (*p* < 0.001, Figure 5). Curcumin showed potential antiproliferative effects in SK-MEL-1 cells.

### 3.7 Curcumin inhibits SK-MEL-1 cell migration

**FIGURE 6:**
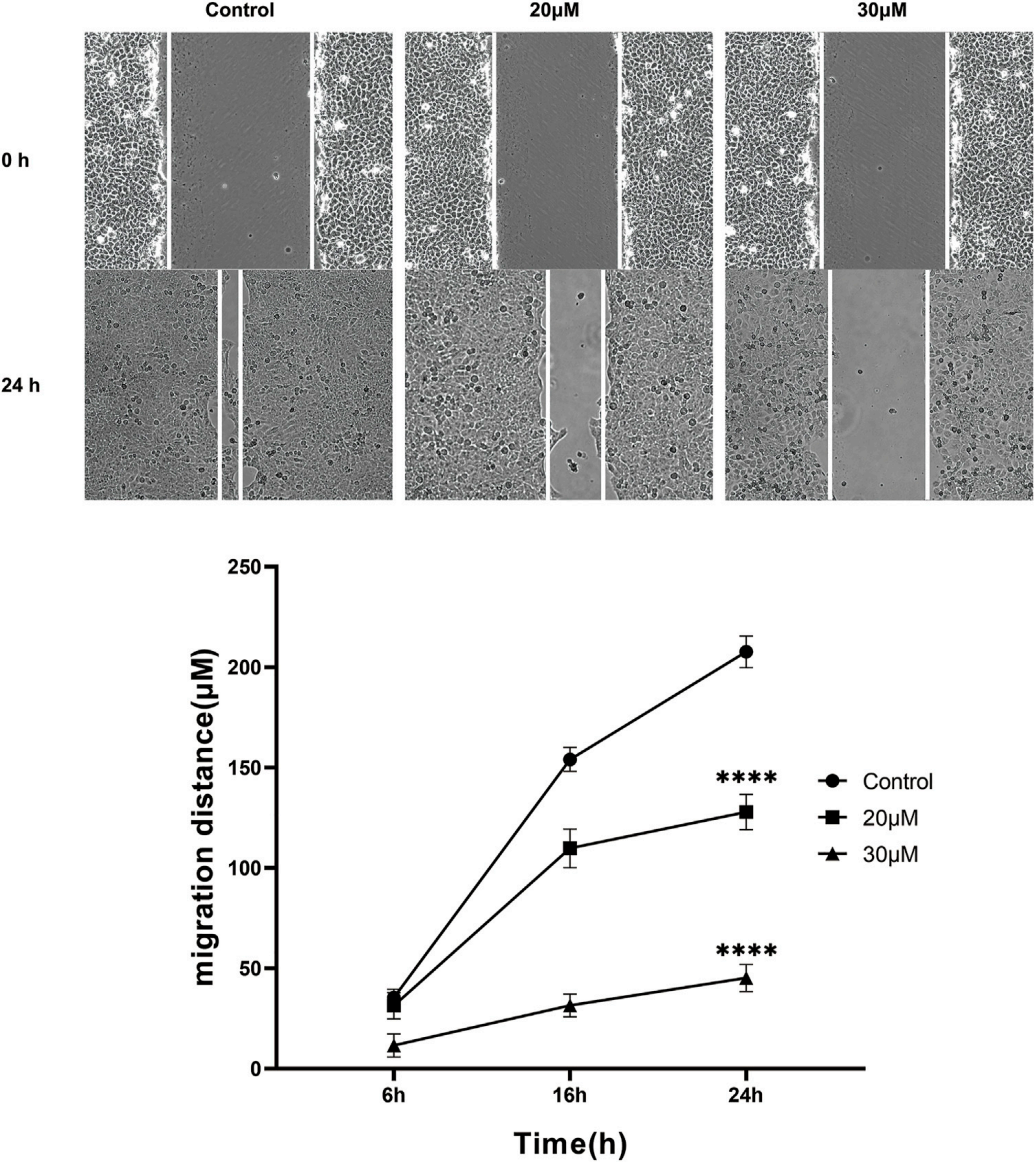
Cell migration evaluation by scratch wound assay. The SK-MEL-1 cells were treated with curcumin in concentrations of 0, 20, and 30 μM. Cell migration was significantly suppressed in curcumin-treated groups in comparison to that of the control group. **p < 0.01 and ***p < 0.001, two-way ANOVA.

The anti-migration effect of curcumin on SK-MEL-1 cells was measured *in vitro* by performing the scratch wound assay. The results (Figure 6) showed that there was no significant difference at 6 h (*p* > 0.05) in terms of migration distance observed among the 3 groups. A concentration of 100 μM curcumin significantly suppresses the migration of SK-MEL-1 cells at 16 h (*p* < 0.01) and 24 h (*p* < 0.001) in comparison to the control group. In addition, compared with the control group, curcumin at a concentration of 50 μM significantly inhibited the migration of SK-MEL-1 cells at 24 h (*p* < 0.01). The results indicated that curcumin had a potential anti-migratory effect in SK-MEL-1 cells.

### 3.8 Curcumin promotes apoptosis in SK-MEL-1 cells

**FIGURE 7:**
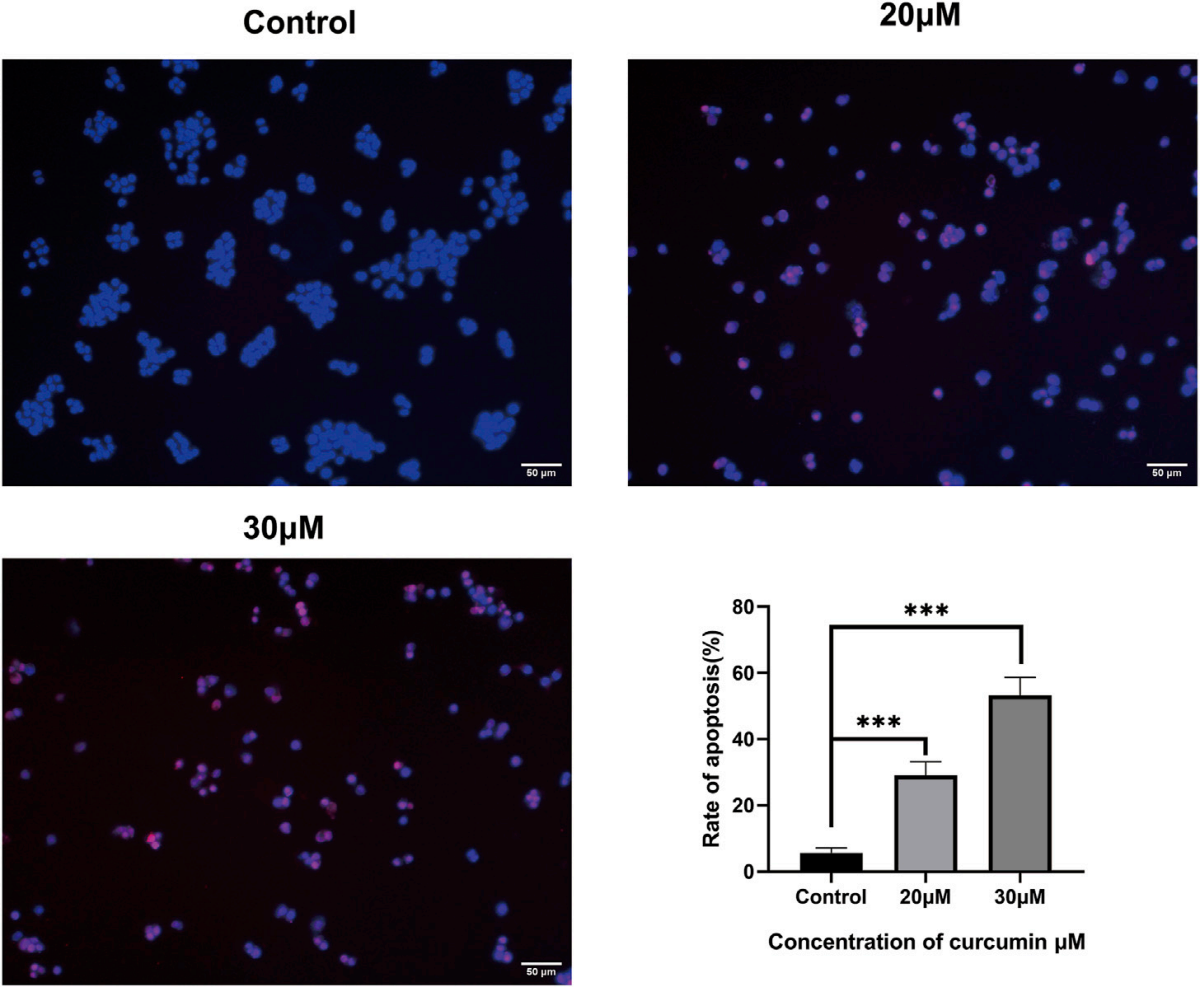
Evaluation of cell apoptosis by TUNEL assay. The SK-MEL-1 cells were treated with curcumin in concentrations of 0, 20, and 30 μM. Cell apoptosis was significantly promoted in curcumin-treated groups in comparison to that of the control group. TUNEL-positive cells were stained in red color, and the nucleus stained by DAPI was in blue. *p < 0.05 and ****p < 0.0001, one-way ANOVA.

**FIGURE 8:**
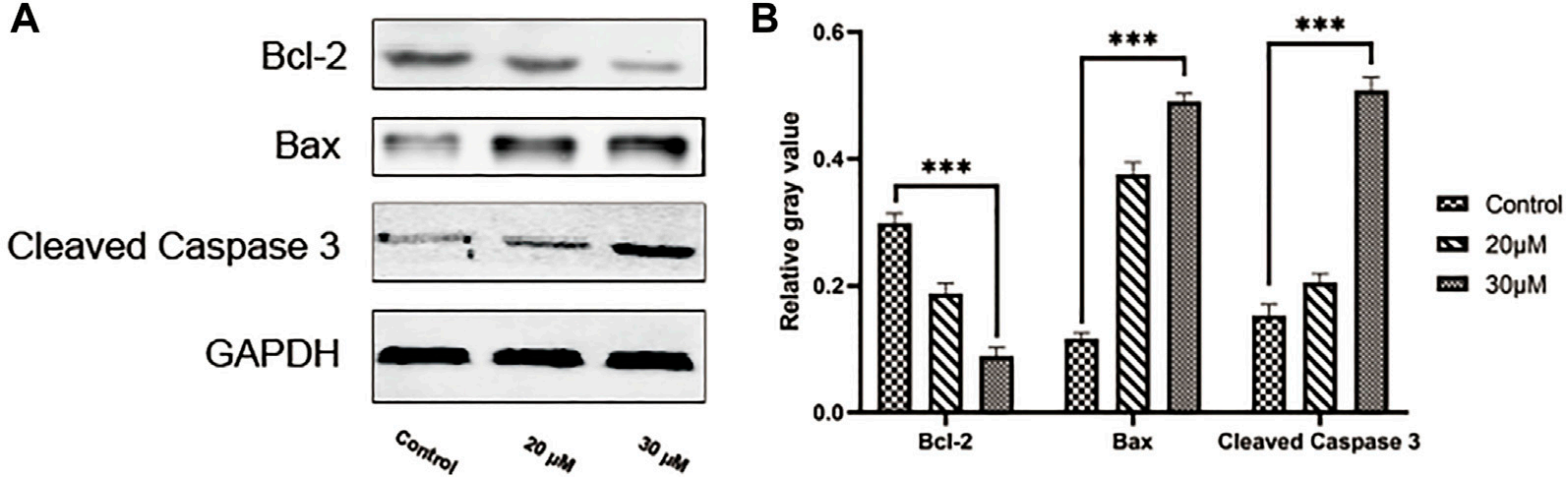
Evaluation of cell apoptosis by western blot. Blots showing proteins in the SK-MEL-1 cells treated with curcumin in different concentrations **(A)** and quantification **(B)**. **p < 0.01, ***p < 0.001 and ****p < 0.0001, one-way ANOVA.

We conducted a TUNEL assay to evaluate the pro-apoptosis effect of curcumin on SK-MEL-1 cells. The results of TUNEL (Figure 7) showed that the apoptosis rate and the apoptosis rate of SK-MEL-1 cells in the curcumin-treated group were significantly higher than those in the control group (*p* < 0.0001). The results of the western blot were consistent with that of the TUNEL assay (Figure 8).

## 4 Discussion

From the point of view of molecular biological networks, traditional Chinese medicine network pharmacology provides a systematic research method in which the application of available traditional Chinese medicine compounds in various diseases can be evaluated. Previous research has shown that curcumin has great potential in preventing and treating various cancers (Ghalaut et al., 2012; Golombick et al., 2012; Ryan et al., 2013). In this study, we demonstrated that the molecular targets of curcumin on SKCM cells can be used as biomarkers of diagnosis and prognosis. In addition, the possible targets and concentrations of curcumin on SKCM were also analyzed. Analysis of clinical survival data has indicated that the survival rate was higher in SKCM patients with a high expression of *DYPD*, *DYPS*, *LYN*, *PRKCQ,* and *TLR1*, while the survival rate was lower in patients with a high expression of *GPI*, *MMP2,* and *PCSK9.* In the detection of tumor tissue and normal tissue, it was shown that the expression of *GPI*, *LYN,* and *TLR1* was enhanced, whereas the expression of *MMP2* was reduced. The targets of curcumin in the treatment of SKCM discovered in this study can be used as prognostic features and provide a theoretical basis for curcumin in the treatment of SKCM.

Co-expression analysis has indicated that these genes were not highly co-expressed with one another at the gene as well as protein levels. However, the regulation of these genes can influence the expression of *TP53* at both gene and protein levels. *TP53* has an important role as a tumor suppressor gene in humans, associated with the induction or inhibition of the cell cycle regulation, apoptosis regulation, DNA repair, and cell senescence-related gene expression after activation (Giaccia and Kastan, 1998). In subsequent *in vitro* experiments, we found that curcumin could upregulate the expression of Caspase3 and Bax in SK-MEL-1 cells and significantly reduce Bcl-2’s expression. Thus, *TP53* may serve as the therapeutic target of curcumin in SKCM treatment. In addition, the GO analysis showed that these target genes were significantly enriched in the regulation of the apoptotic process, positive regulation of cell death, and regulation of the macromolecule metabolic process. The results of the KEGG analysis indicated that curcumin may play a role in SKCM by regulating metabolic pathways in tumor-related signaling pathways and the biosynthesis of amino acids and proteoglycans.

Based on GSEA analysis and literature review, we believe that these important genes/proteins are closely related to the occurrence of SKCM. *DYPD* can affect SKCM by regulating the cell response to UV, MAPK signaling pathway, JAK-STAT signaling pathway, apoptosis signaling pathway, and affecting cell-to-cell adhesion. *DPYD* is a vital enzyme in the metabolic pathway and is related to the drug response to 5-fluorouracil chemotherapy (Kristensen et al., 2010; Kimura et al., 2011). In addition, GPI function by regulating UV, tumor metastasis, and NF-κB signaling pathways. *GPI* is a glycolysis enzyme that plays a biological role through cell secretion. Its overexpression has been related to increased invasive phenotypes and mortality in many types of cancer (Lyu et al., 2016; Ma et al., 2018). Therefore, the survival rate of patients with elevated *GPI* is decreased, which was also observed in our study. Moreover, *LYN*, *MMP2*, *PRKCQ,* and *TLR1* can affect SKCM by influencing tumor cell metastasis, UV-induced cell injury, the NF-κB signaling pathway, and apoptosis. *LYN* can regulate proliferation, differentiation, migration, and apoptosis, and over-expression of *LYN* plays a vital role in solid tumors (Tabariès et al., 2015; Q. Zhang et al., 2019). Previous research has indicated that *MMP2* can produce the key pre-invasion factor induced by carcinogenic inflammation, and promotes tumor growth and invasion (Winerdal et al., 2018; W. Zhang et al., 2006). *PRKCQ* is widely expressed in the entire hematopoietic system and can induce the production and migration of breast tumors (Vyas et al., 2001; Byerly et al., 2016). *TLR1* mediates local inflammation by affecting NF-κB. In pancreatic ductal carcinoma, the strong expression of *TLR1* indicates a good prognosis, while the negative expression of *TLR1* is a sign of poor prognosis (Lanki et al., 2019). We further investigated the effects of curcumin on the proliferation and apoptosis of human SKCM cells *in vitro*.

The cell wound assay demonstrated inhibited SK-MEL-1 cell growth by curcumin in a dose-dependent manner, and TUNEL staining showed that apoptosis increased significantly. In addition, after treatment with curcumin, the western blot demonstrated that curcumin could stimulate the activation of Caspase3 and downregulate the ratio of BCL2 and Bax. These findings are consistent with previously reported results of curcumin-induced apoptosis in osteosarcoma cells (Khodaei et al., 2022). Living cells possess higher levels of Bcl-2, which leads to the inhibition of apoptosis. Bcl-2 regulates the cellular activities of proteins related to cell proliferation or apoptosis, such as Caspase3 and Bax36. The first stage of the intrinsic apoptotic pathway relies on the activity of Bax, which modulates cellular fidelity to the pathway by altering mitochondrial physiology. Therefore, the effect of apoptosis induction by curcumin in SK-MEL-1 cells can be elucidated by detecting the expression of Caspase3 and the ratio of Bcl-2 to Bax. Thus, these target genes may act together on SKCM and may be potential therapeutic targets in SKCM.

## 5 Conclusion

We applied a network pharmacology method to identify the potential mechanisms of curcumin for the SKCM treatment methods. The common target genes might participate in the regulation of the inflammatory microenvironment of the tumor, thereby affecting the occurrence and metastasis of the tumor and improving the prognosis of SKCM patients. The *in vitro* experiments have indicated that curcumin has anti-proliferative and pro-apoptotic effects in SK-MEL-1 cells. Based on the functions that these hub genes occupy in cell proliferation and apoptosis; further *in vitro* research is necessary to clarify the specific anti-SKCM effect of curcumin.

## Data Availability

The original contributions presented in the study are included in the article/Supplementary Material, further inquiries can be directed to the corresponding author.
