# Supplementary material for: Anti-proliferative and pro-apoptotic effects of curcumin on skin cutaneous melanoma: Bioinformatics analysis and in vitro experimental studies
Source: Front Genet. 2022 Sep 12;13:983943. doi: 10.3389/fgene.2022.983943 (PMC9510772; doi:10.3389/fgene.2022.983943)
Supplement: Supplementary file 2 [file DataSheet1.DOCX]

Supplementary Material

## Supplementary Figures


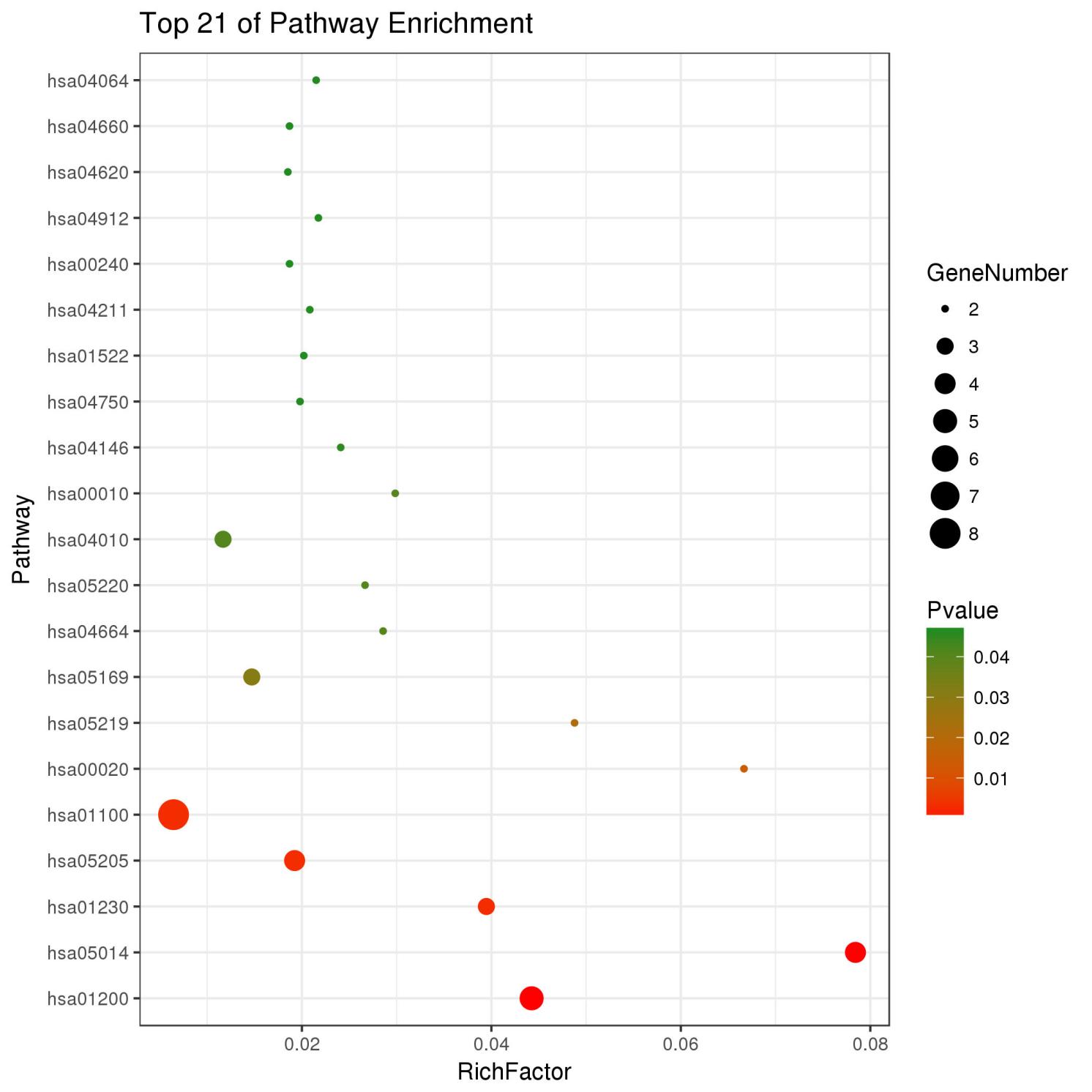


**Supplementary Figure 1.** The Kyoto Encyclopedia of Genes and Genomes analysis.

**
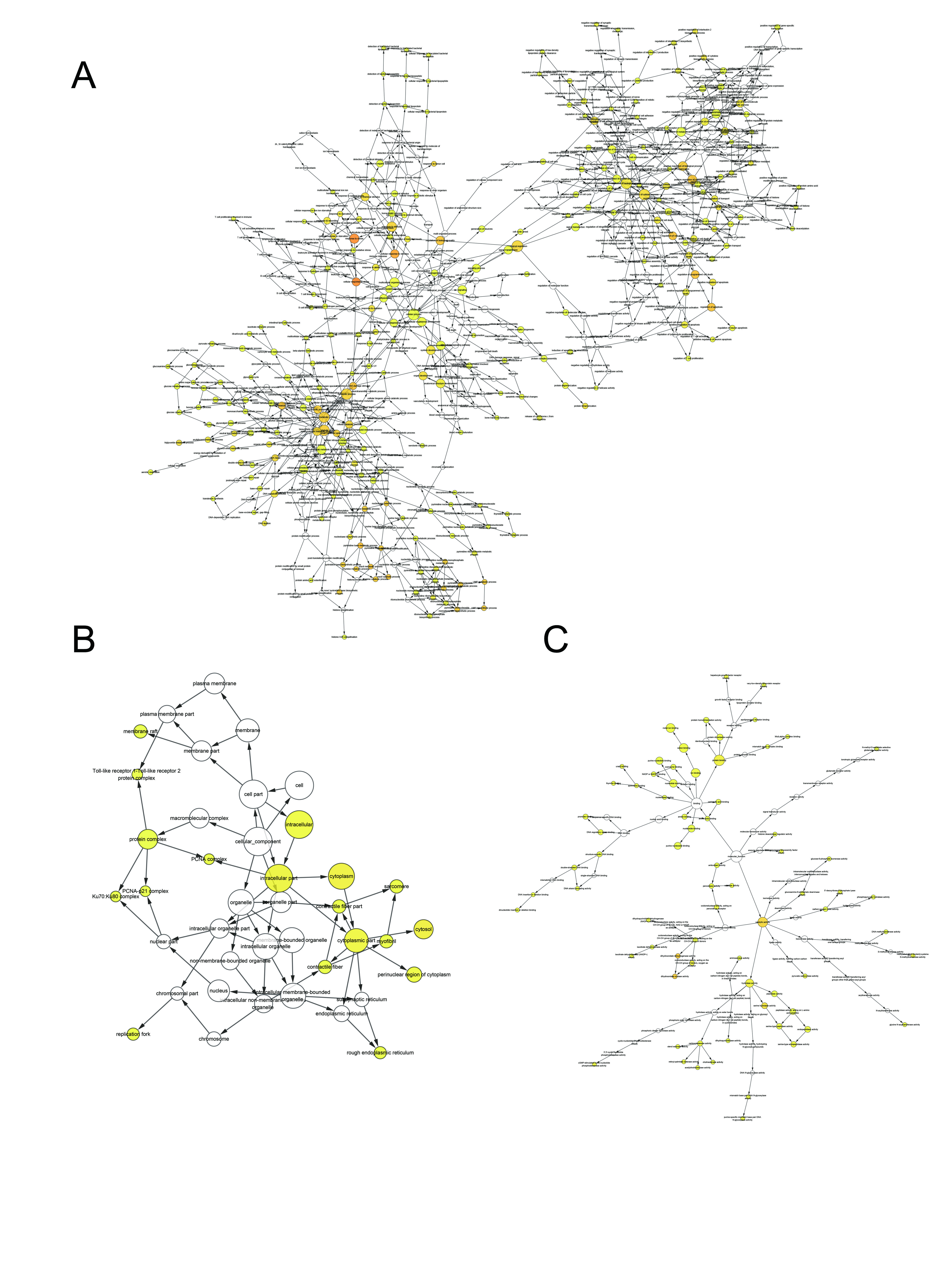
**

**Supplementary Figure 2.** Gene Ontology analysis by BiNGO. (A) BP outcomes; (B) CC outcomes; (C) MF outcomes. BiNGO, Biological networks Gene Ontology; BP, biological process; CC, cellular component; MF, molecular function.


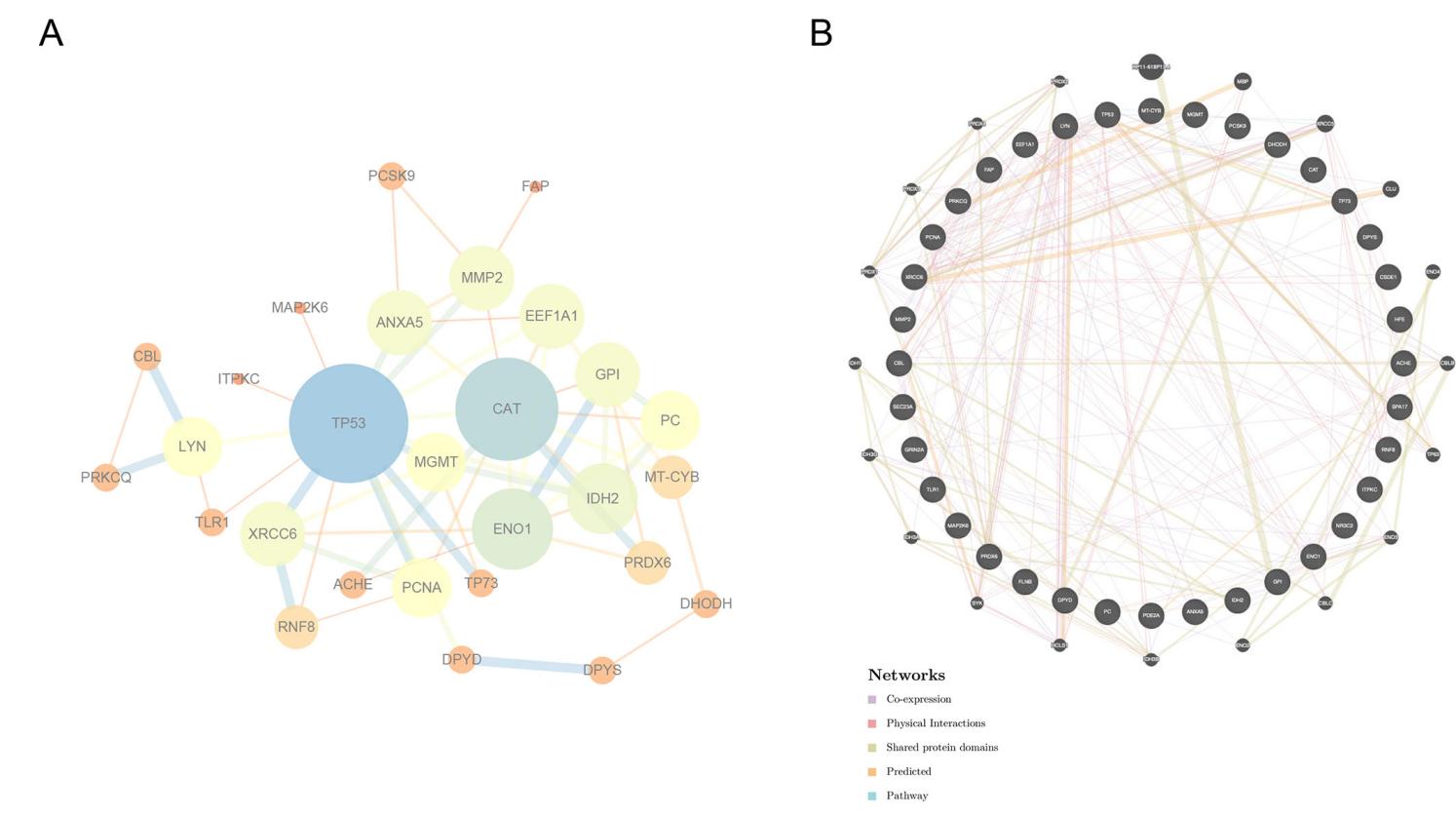


**Supplementary Figure 3.** (A) The PPI network for physical and functional connections of common targets by STRING. The nodes signify proteins and the edges signify the pairs of interactions between proteins. The size of the node and color represent the enriched degree, while the size of the edge and color represent the combined score. (B) The construction of the GI network of common targets by GeneMANIA. The black circles with slash refer to the query genes, and the other genes refer to the predicted ones. PPI, Protein-Protein Interaction; STRING, Search Tool for the Retrieval of Interacting Genes/Proteins; GI, Genetic Interaction.


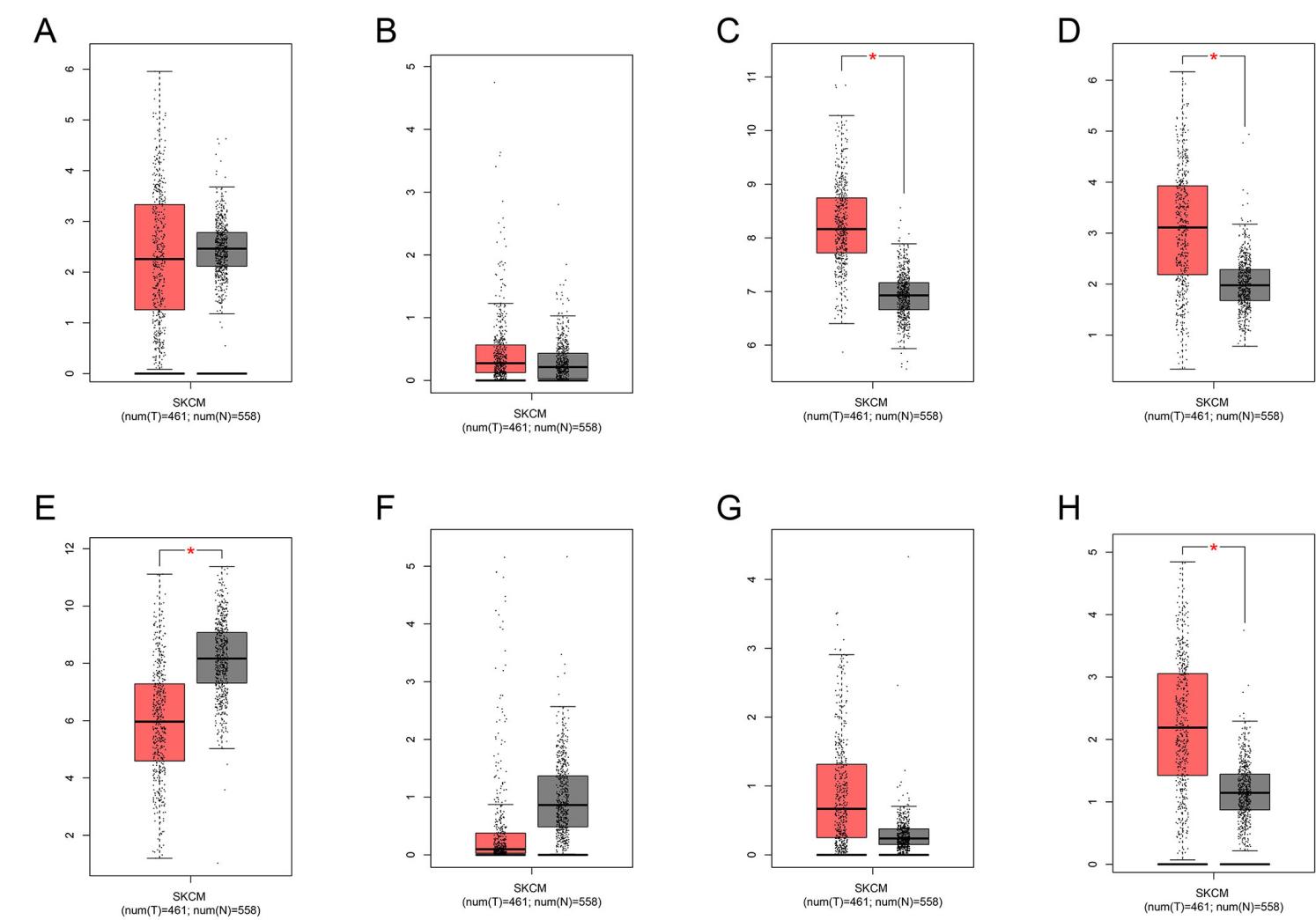


**Supplementary Figure 4.** The boxplots for the expression of hub genes both in normal tissues and SKCM tissues by using GEPIA. (A) Boxplot for *DPYD* expression; (B) boxplot for *DPYS* expression; (C) boxplot for *GPI* expression; (D) boxplot for *TLR1* expression; (E) boxplot for *MMP2* expression; (F) boxplot for *PCSK9* expression. (G) boxplot for *PRKCQ* expression; (H) boxplot for *LYN* expression. GEPIA, Gene Expression Profiling Interactive Analysis.


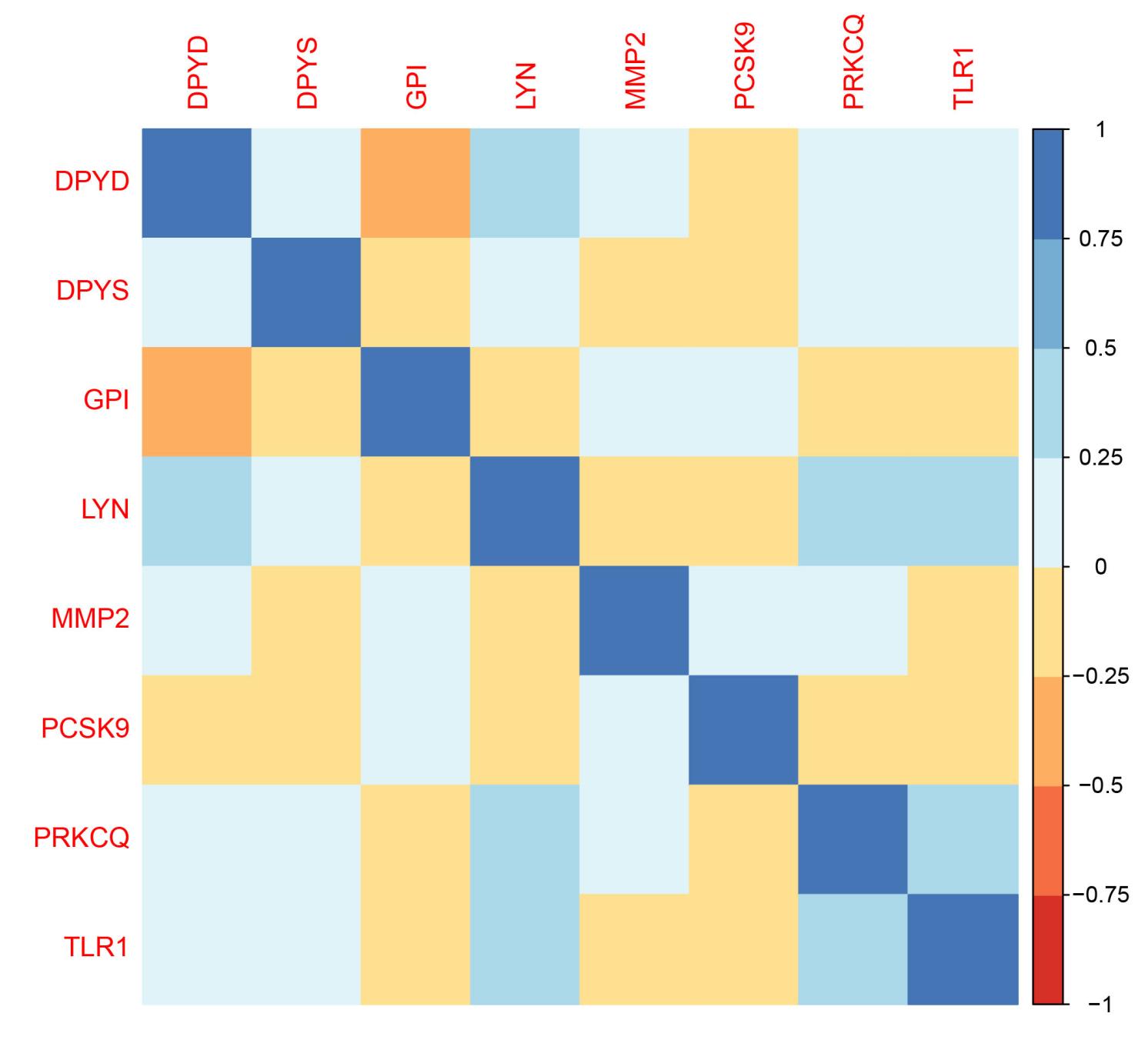


**Supplementary Figure 5.** Pearson correlation coefficients of the expression levels of hub genes.


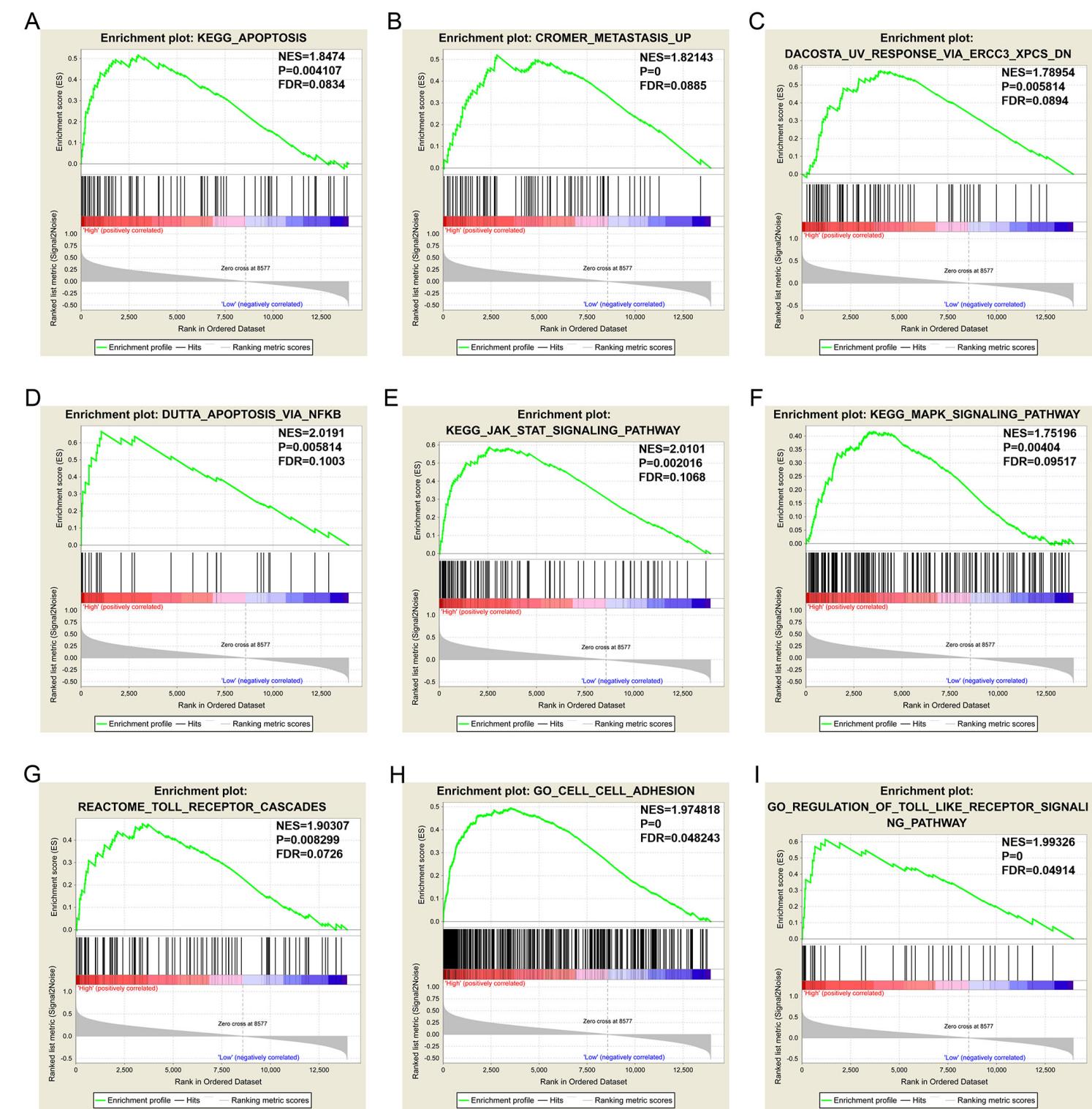


**Supplementary Figure 6.** Enrichment plots of the Gene Set Enrichment Analysis (GSEA) for *DYPD*.


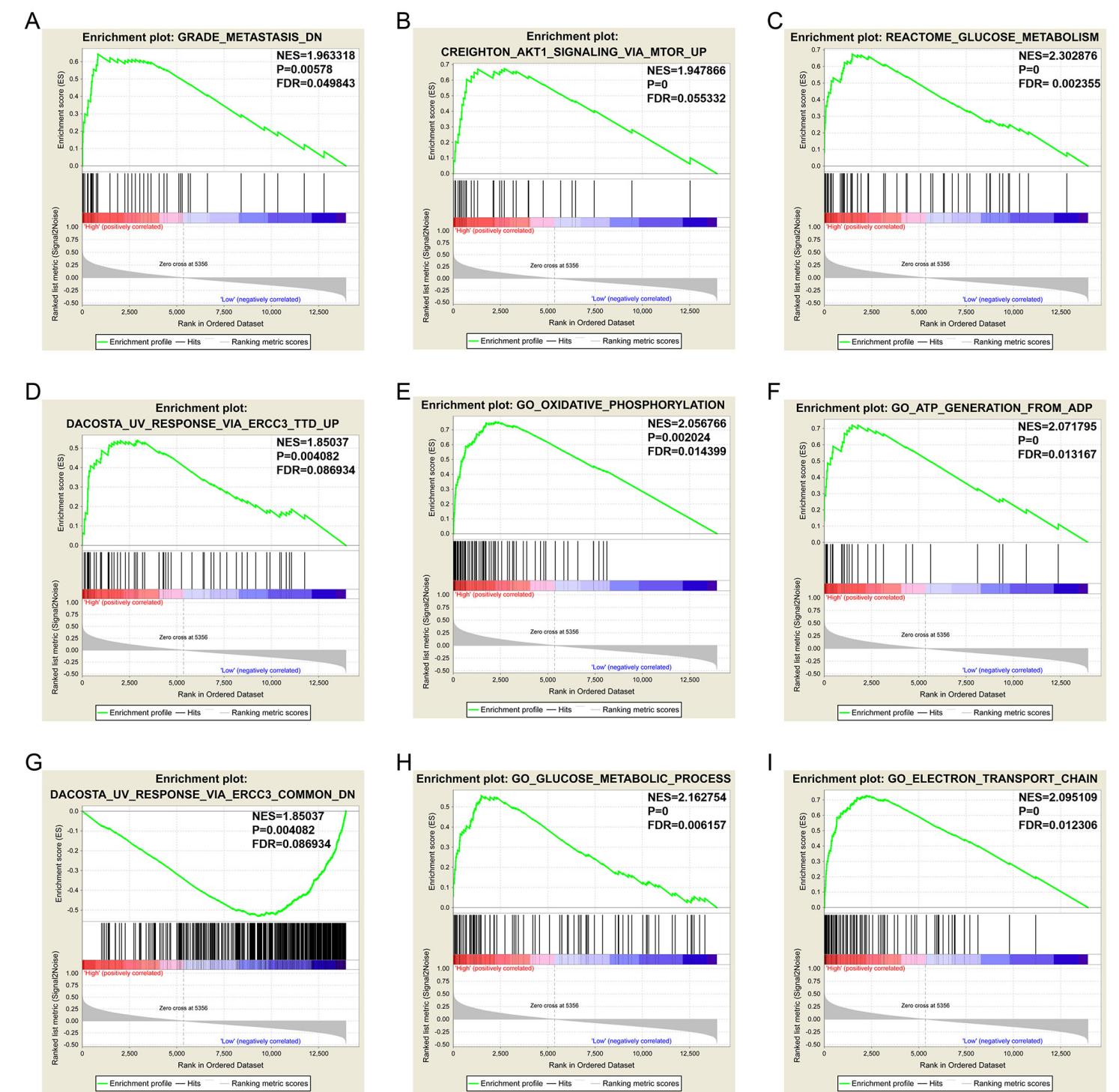


**Supplementary Figure 7.** Enrichment plots of the Gene Set Enrichment Analysis (GSEA) for *GPI*.


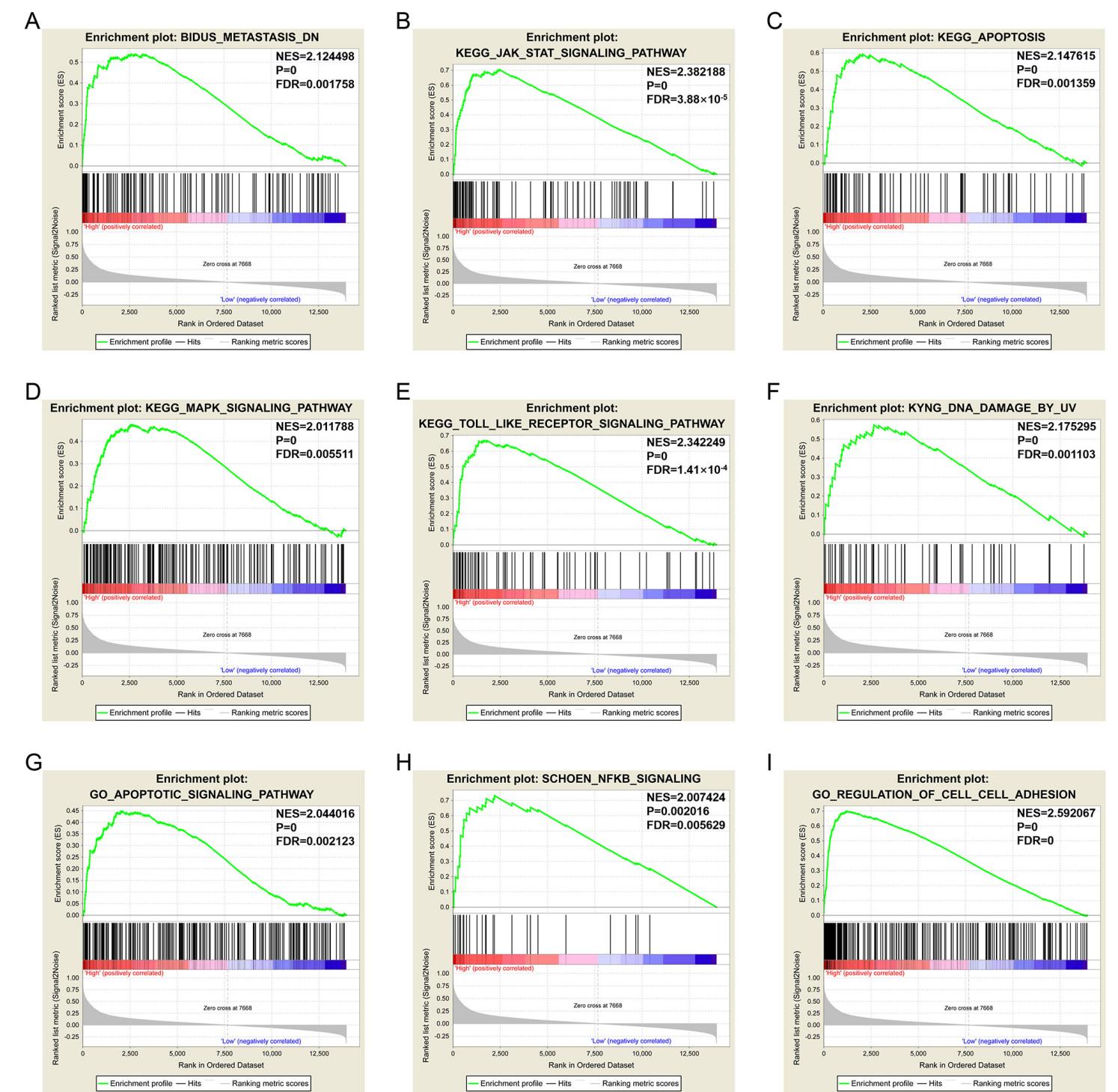


**Supplementary Figure 8.** Enrichment plots of the Gene Set Enrichment Analysis (GSEA) for *LYN*.


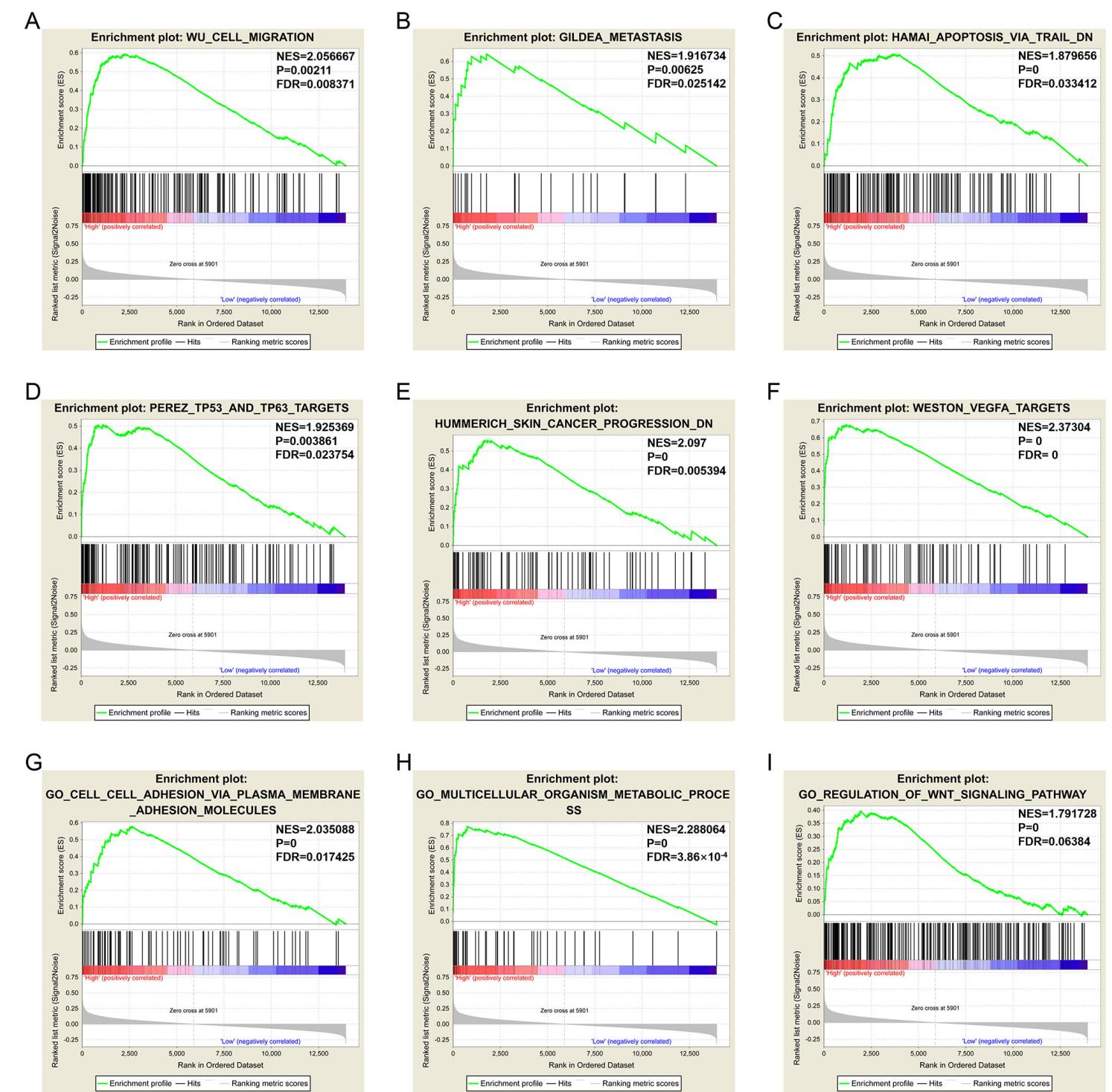


**Supplementary Figure 9.** Enrichment plots of the Gene Set Enrichment Analysis (GSEA) for *MMP2*.


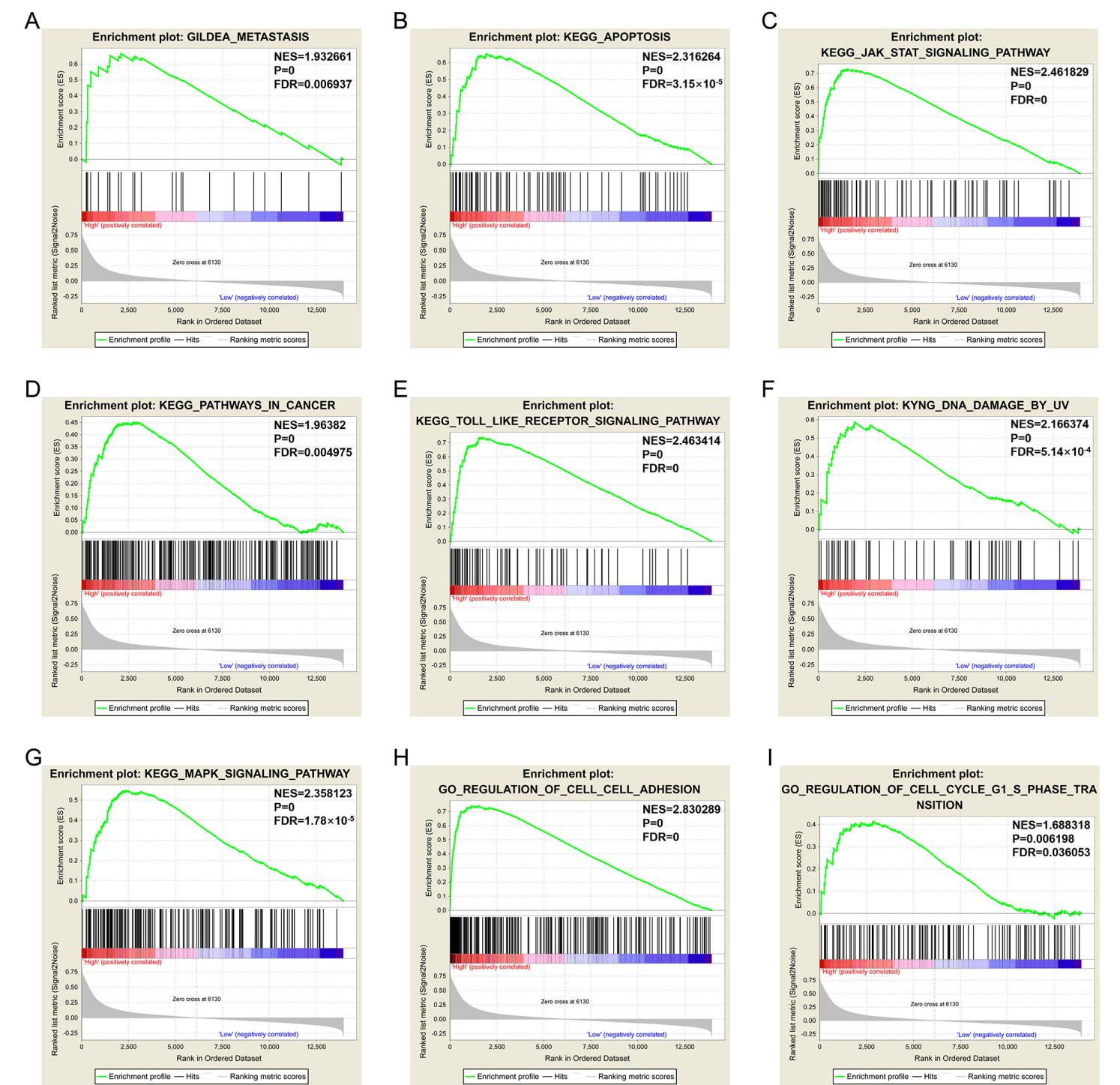


**Supplementary Figure 10.** Enrichment plots of the Gene Set Enrichment Analysis (GSEA) for *PRKCQ*.


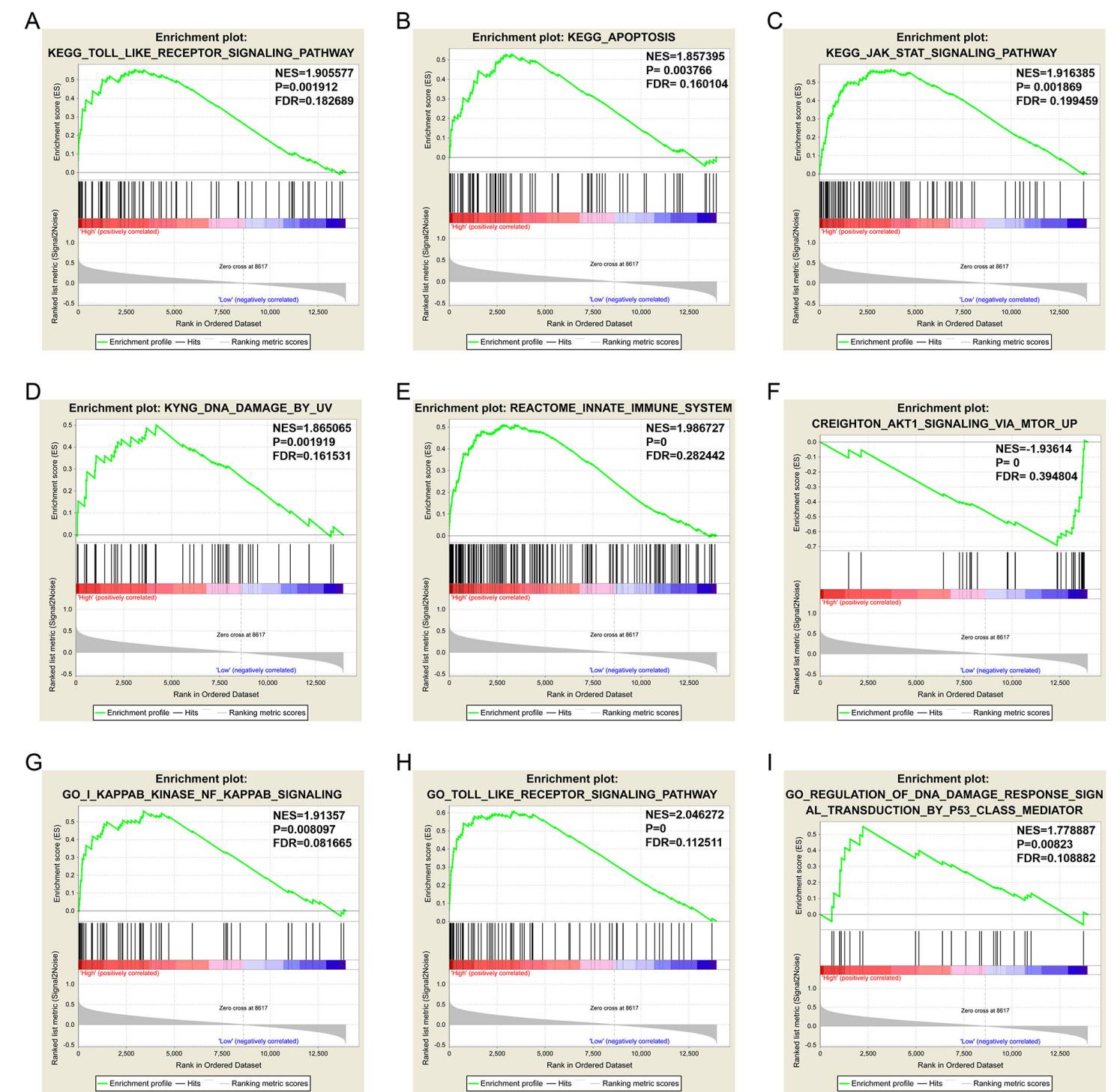


**Supplementary Figure 11.** Enrichment plots of the Gene Set Enrichment Analysis (GSEA) for *TLR1*.

## Supplementary Tables

**Supplementary Table 1.** Gene Ontology analysis results.

| Term | ID | Ratio | Corrected P-Value |
| --- | --- | --- | --- |
| cytoplasm | GO:0005737 | 0.00285959 | 1.49E-07 |
| catabolic process | GO:0009056 | 0.00843882 | 1.49E-07 |
| cytoplasmic part | GO:0044444 | 0.00340737 | 1.92E-07 |
| organic substance catabolic process | GO:1901575 | 0.00835189 | 4.33E-07 |
| cytosol | GO:0005829 | 0.00525547 | 4.35E-06 |
| cellular catabolic process | GO:0044248 | 0.00855826 | 4.35E-06 |
| single-organism metabolic process | GO:0044710 | 0.00478589 | 4.35E-06 |
| response to endogenous stimulus | GO:0009719 | 0.00840336 | 4.35E-06 |
| response to stress | GO:0006950 | 0.00492341 | 7.54E-06 |
| protein binding | GO:0005515 | 0.00262738 | 9.05E-06 |
| regulation of apoptotic process | GO:0042981 | 0.00865801 | 1.00E-05 |
| regulation of programmed cell death | GO:0043067 | 0.00858369 | 1.01E-05 |
| response to external stimulus | GO:0009605 | 0.00664452 | 1.15E-05 |
| catalytic activity | GO:0003824 | 0.00375402 | 1.53E-05 |
| regulation of cell death | GO:0010941 | 0.00806994 | 1.58E-05 |
| intracellular part | GO:0044424 | 0.00222866 | 1.81E-05 |
| intracellular | GO:0005622 | 0.0021725 | 3.56E-05 |
| response to organic substance | GO:0010033 | 0.00533239 | 3.65E-05 |
| extracellular space | GO:0005615 | 0.00825206 | 3.65E-05 |
| binding | GO:0005488 | 0.00214792 | 3.65E-05 |
| organelle | GO:0043226 | 0.00227844 | 3.65E-05 |
| response to stimulus | GO:0050896 | 0.00295494 | 3.65E-05 |
| response to organonitrogen compound | GO:0010243 | 0.01130653 | 3.65E-05 |
| positive regulation of apoptotic process | GO:0043065 | 0.01405975 | 3.65E-05 |
| positive regulation of programmed cell death | GO:0043068 | 0.01393728 | 3.65E-05 |
| single-organism process | GO:0044699 | 0.00226811 | 3.65E-05 |
| plasma membrane | GO:0005886 | 0.00388548 | 3.65E-05 |
| single-organism catabolic process | GO:0044712 | 0.01115242 | 3.65E-05 |
| cellular metabolic process | GO:0044237 | 0.00263425 | 4.02E-05 |
| membrane-bounded organelle | GO:0043227 | 0.00236467 | 4.27E-05 |
| enzyme binding | GO:0019899 | 0.00684151 | 4.27E-05 |
| cell periphery | GO:0071944 | 0.00380457 | 4.27E-05 |
| plasma membrane part | GO:0044459 | 0.00554236 | 4.27E-05 |
| positive regulation of cell death | GO:0010942 | 0.01315789 | 4.40E-05 |
| regulation of macromolecule metabolic process | GO:0060255 | 0.00353482 | 4.99E-05 |
| apoptotic process | GO:0006915 | 0.00668524 | 4.99E-05 |
| programmed cell death | GO:0012501 | 0.00660429 | 5.52E-05 |
| response to drug | GO:0042493 | 0.0162037 | 5.72E-05 |
| response to chemical | GO:0042221 | 0.00420584 | 5.76E-05 |
| cellular response to endogenous stimulus | GO:0071495 | 0.0085034 | 5.91E-05 |
| response to nitrogen compound | GO:1901698 | 0.00995575 | 6.36E-05 |
| organic substance metabolic process | GO:0071704 | 0.00252403 | 7.37E-05 |
| cellular component organization | GO:0016043 | 0.00339616 | 8.06E-05 |
| cell death | GO:0008219 | 0.00624025 | 8.27E-05 |
| cellular process | GO:0009987 | 0.00203943 | 8.27E-05 |
| multi-organism process | GO:0051704 | 0.00552251 | 9.80E-05 |

Ratio: Input number / Background number.

**Supplementary Table 2.** Correlation of expression between hub genes.

|  | DPYD | DPYS | GPI | LYN | MMP2 | PCSK9 | PRKCQ | TLR1 |
| --- | --- | --- | --- | --- | --- | --- | --- | --- |
| DPYD | 1 | 0.110^*^ | -0.289^**^ | 0.400^**^ | 0.005 | -0.123^**^ | 0.237^**^ | 0.239^**^ |
| DPYS | 0.110^*^ | 1 | -0.107^*^ | 0.058 | -0.036 | -0.054 | 0.040 | 0.020 |
| GPI | -0.289^**^ | -0.107^*^ | 1 | -0.200^**^ | 0.061 | 0.167^**^ | -0.083 | -0.230^**^ |
| LYN | 0.400^**^ | 0.058 | -0.200^**^ | 1 | -0.063 | -0.108^*^ | 0.472^**^ | 0.323^**^ |
| MMP2 | 0.005 | -0.036 | 0.061 | -0.063 | 1 | 0.135^**^ | 0.065 | -0.073 |
| PCSK9 | -0.123^**^ | -0.054 | 0.167^**^ | -0.108^*^ | 0.135^**^ | 1 | -0.026 | -0.098^*^ |
| PRKCQ | 0.237^**^ | 0.040 | -0.083 | 0.472^**^ | 0.065 | -0.026 | 1 | 0.380^**^ |
| TLR1 | 0.239^**^ | 0.020 | -0.230^**^ | 0.323^**^ | -0.073 | -0.098^*^ | 0.380^**^ | 1 |

**: The correlation was significant at a 0.01 level.

*: The correlation was significant at a 0.05 level.
