# Supplementary material for: Anti-proliferative and pro-apoptotic effects of curcumin on skin cutaneous melanoma: Bioinformatics analysis and in vitro experimental studies
Source: Front Genet. 2022 Sep 12;13:983943. doi: 10.3389/fgene.2022.983943 (PMC9510772; doi:10.3389/fgene.2022.983943)
Supplement: Supplementary file 1 [file DataSheet2.DOCX]

**Response to Reviewers Point-by-Point**

Dear Editors and Reviewers:

Thank you for your letter and for the reviewers’ comments concerning our manuscript entitled **“Anti-proliferative and pro-apoptotic effects of curcumin on skin cutaneous melanoma: bioinformatics analysis and in vitro experimental studies” (ID:** **983943).** Those comments are all valuable and very helpful for revising and improving our paper, as well as the important guiding significance to our researches. We have studied comments carefully and have made correction which we hope meet with approval. Revised portion are marked in the paper. The main corrections in the paper and the responds to the reviewer’s comments are as flowing:

**Reviewer 1**

The article manuscript used data from the Cancer Genome Atlas and the TCM Systematic Pharmacology database as sources to search for SKCM and curcumin-related information, using as well as identifying new prognostic biomarkers for SKCM. The article manuscript makes good use of this means of data discovery and adds in vitro experimental validation to the subsequent analysis, which has a good article structure pulse and is recommended for publication; however, the same drawback of weak experimental validation evidence exists.

**Response:**

Thank you for your nice and positive comments.

1. There is an error in lines 145-148 of the manuscript, where the meaning of "ix" is unclear.

**Response:**

Thanks for your careful comments.

We apologize for the error regarding "ix" here and have made corrections for the error that should have been made. Please see the **revised Manuscript (lines 145-148).**

2. In Figure 1 of the manuscript, an arrow should be added to the flow chart to indicate the flow direction of the article.

**Response:**

Thanks for your valuable comments.

We have modified the figure as per your request and added arrows in the revised Figure 1 to indicate the flow direction. Please see the **Figure 1-Revised.**

3. Please identify the URL of TCMSP (http://lsp.nwu.edu.cn/tcmsp.php) in the article, I tried to open the database but to no avail.

**Response:**

Thanks for your careful comments.

We appreciate your verifying the validity of the TCMSP website and pointing out our error; in fact, the domain name of this website changes frequently, and at this moment the address of this website is: https://old.tcmsp-e.com/tcmsp.php. Please see the **revised Manuscript (lines 77 and 380).**

**Reviewer 2**

In this manuscript "Anti-proliferative and pro-apoptotic effects of curcumin on skin cutaneous melanoma: bioinformatics analysis and in vitro experimental studies" the authors justify the efficacy of curcumin. There is no novelty for this study. Curcumin is a very widely studied molecule and several reports have shown its potential. Hence the work lacks novelty. It would have been ideal if the authors also show the mode of action. Does it have a role in oxidataive stress?

**Response:**

Thank you for your nice and positive comments.

The authors have not demonstrated any mechanisms or done any functional experiments.

**Response:**

Thanks for your comments.

In fact, in this study, we first analyzed and determined the pathways and target genes of curcumin in the treatment of cutaneous melanoma (SKCM) by means of bioinformatics, furthermore, the effects of varying concentrations of curcumin on proliferation, migration, and apoptosis of SKCM cells were assessed in vitro. In fact, the proliferation, cell migration and apoptosis assays of SK-MEL-1 cells in vitro are the most appropriate functional assays. Please see the **revised Manuscript (lines 259-280).**

The authors need to demonstrate the role of Curcumin on cell cycle arrest and its affect, on normal tissue/cells.

**Response:**

Thanks for your valuable comments.

Curcumin and its derivatives induced ROS production, leading to DNA breaks, chromatin condensation and nuclear atrophy, and significantly increased apoptotic cells and inhibited classical signalling pathways, including nuclear factor kappa B (NF-κB), signal transducer and activator of transcription 3 (STAT3), Akt / Mtor and Wnt / β-catenin, inducing endoplasmic reticulum stress in SKCM, and reactive oxygen species (ROS) expression thus enhancing oxidative stress damage. (**PMID: 18493855, Apoptosis . 2008 Jul;13(7):904-14; PMID: 28926928, Biomed Pharmacother . 2017 Nov;95:1177-1186; PMID:16007726, Cancer . 2005 Aug 15;104(4):879-90; PMID: 25730045, Genet Mol Res . 2015 Feb 6;14(1):1056-67; PMID: 15179184, Melanoma Res . 2004 Jun;14(3):165-71; PMID: 34572508, Biomolecules . 2021 Aug 31;11(9):1295; PMID: 29673545, Nutr Res . 2018 Mar;51:67-81).**

Please see the **revised** **Manuscript (lines 58-62).**

We tried our best to improve the manuscript and made some changes in the manuscript. These changes will not influence the content and framework of the paper.

We appreciate for Editors/Reviewers’ warm work earnestly, and hope that the correction will meet with approval.

Once again, thank you very much for your comments and suggestions.
